# Antivenoms for Snakebite Envenoming: What Is in the Research Pipeline?

**DOI:** 10.1371/journal.pntd.0003896

**Published:** 2015-09-10

**Authors:** Emilie Alirol, Pauline Lechevalier, Federica Zamatto, François Chappuis, Gabriel Alcoba, Julien Potet

**Affiliations:** 1 Médecins Sans Frontières, London, United Kingdom; 2 Médecins Sans Frontières, Paris, France; 3 Médecins Sans Frontières, Geneva, Switzerland; 4 University Hospitals of Geneva, Geneva, Switzerland; 5 Médecins Sans Frontières Access Campaign, Geneva, Switzerland; University of Kelaniya, SRI LANKA

## Introduction

Of the 24 neglected tropical diseases (NTDs) and conditions listed by WHO, snakebite is among the top killers [1]. Tens of thousands of people die each year as a result of snakebite envenoming, with close to 50,000 deaths in India alone [2] and up to 32,000 in sub-Saharan Africa [3]. Yet there are few sources of effective, safe, and affordable antivenoms. The regions that bear the highest snakebite burden are especially underserved [4].

The Fav-Afrique antivenom, produced by Sanofi Pasteur (France), is considered safe and effective and is one of the few antivenoms to be approved by a Stringent Regulatory Authority (French National Regulatory Authority), although limited formal evidence has been published [5,6]. It is polyvalent, targeting most of the medically important snake species in sub-Saharan Africa. In particular, it is highly effective in treating envenoming by *Echis ocellatus*, the West African saw-scaled viper [5–7] that causes great morbidity and mortality throughout the West and Central African savannah. The venom of *E*. *ocellatus* may induce systemic haemorrhage, coagulopathy, and shock, as well as extensive local tissue damage. In the absence of treatment, the case fatality rate is 10%–20% [8]. Médecins Sans Frontières (MSF) uses Fav-Afrique in its projects in sub-Saharan Africa, notably in Paoua in Central African Republic (CAR), where *E*. *ocellatus* envenoming is frequent [9]. Worryingly, MSF has been informed that the production of Fav-Afrique by Sanofi Aventis will be permanently discontinued. The last batch was released in January 2014, with an expiry date of June 2016. All the vials produced have already been sold by Sanofi Pasteur.

Although several alternative antivenom products target a similar list of species as Fav-Afrique, there is currently no evidence of their safety and effectiveness. We aimed to review the evidence for the efficacy and safety of existing and in-development snake antivenoms, and to list the alternatives to Fav-Afrique in sub-Saharan Africa.

## Search Strategy

We searched clinical trial registries (National Institutes of Health clinicaltrials.gov and WHO International Clinical Trial Registry Platform) and a publication database (EMBASE) to identify ongoing and completed clinical trials. The registries were searched by condition using the keywords “snakebite” OR “snake bite” OR “snake envenom*” OR “envenom*” OR “bite.” Publication database search strategy was based on the Medical Subject Heading (MeSH) terms “clinical trial” AND “snake bites” AND “polyclonal antiserum OR snake venom antiserum OR venom antiserum.” All terms were explored, and results were limited to studies conducted in humans. No time limits were imposed. Searches were conducted in September 2014 and included all records from the launch of the databases. Only those studies with a design compatible with that of a clinical trial (prospective, comparative, and interventional) and with the definition given by the CONSORT glossary were included. Prospective, single-arm cohorts were not considered as clinical trials.

## Search Results

The registry searches yielded 29 records, four of which were observational studies. Among the interventional studies, 12 investigated antivenom as an intervention (eight were retrieved out of 176,201 records in clinicaltrials.gov and 12 out of 254,285 in ICTRP). Table 1 summarises the characteristics of the 12 trials. Four trials were sponsored by pharmaceutical companies and the remainder, by an individual researcher or academic institution. Four trials were open for recruitment and five were completed or terminated. A total of 11 different antivenoms were being investigated, most in only one trial.

**Table 1:** List of clinical trials investigating snake antivenom published in clinical trials registries.

Trial ID number	Title	Sponsor	Type of funding	Location	Year of trial registration	Recruitment status	Results published
NCT00303303	The Efficacy of Crotaline Fab Antivenom for Copperhead Snake Envenomations	Carolinas Healthcare System	Government	United States	2006	Terminated	No
NCT00636116	Phase 3 Multicenter Comparative Study to Confirm Safety and Effectiveness of the F(ab)2 Antivenom Anavip	Instituto Bioclon S.A. de C.V.	Industry	US	2008	Completed	No
NCT00639951	Study to Evaluate the Efficacy of Two Treatment Schemes With Antivipmyn for the Treatment of Snake Bite Envenomation	Instituto Bioclon S.A. de C.V.	Industry	Mexico	2008	Recruiting	NA
NCT00811239	A Controlled Clinical Trial on The Use of a Specific Antivenom Against Envenoming by *Bungarus Multicinctus*	Hanoi Medical University	Government	Vietnam	2008	Completed	Yes [21]
NCT00868309	A Comparison of Crotalinae (Pit Viper) Equine Immune F(ab)2 Antivenom (Anavip) and Crotalidae Polyvalent Immune Fab, Ovine Antivenom (CroFab) in the Treatment of Pit Viper Envenomation	Instituto Bioclon S.A. de C.V.	Industry	US	2008	Completed	Yes [22]
ISRCTN01257358	Clinical trial of two new anti-snake venoms for the treatment of patients bitten by poisonous snakes in Nigeria	Nigeria MoH	Unknown	Nigeria	2009	Completed	Yes [23]
SLCTR/2010/006	Low dose versus high dose of Indian polyvalent snake antivenom in reversing neurotoxic paralysis in common krait (*Bungarus caeruleus*) bites: an open labelled randomised controlled clinical trial in Sri Lanka	Individual researcher	None	Sri Lanka	2010	Not recruiting	No
ACTRN12611000588998	A randomised controlled trial of antivenom and corticosteroids for red-bellied black snake envenoming	Individual researcher	Government	Australia	2011	Not recruiting	No
NCT01284855	Comparison of Two Dose Regimens of Snake Antivenom for the Treatment of Snake Bites Envenoming in Nepal	University of Geneva	Government	Nepal	2011	Not recruiting	No
NCT01337245	Emergency Treatment of Coral Snake Envenomation With Antivenom	University of Arizona	Government	US	2011	Recruiting	NA
ACTRN12612001062819	A randomized controlled trial (RCT) of a new monovalent antivenom (ICP Papuan taipan antivenom) for the treatment of Papuan taipan (*Oxyuranus scutellatus*) envenoming in Papua New Guinea	University of Melbourne	Government	Papua New Guinea	2012	Recruiting	NA
NCT01864200	A Randomized, Double-Blind, Placebo-Controlled Study Comparing CroFab Versus Placebo With Rescue Treatment for Copperhead Snake Envenomation (Copperhead RCT)	BTG International Inc.	Industry	US	2013	Recruiting	NA

**Fig 1:**
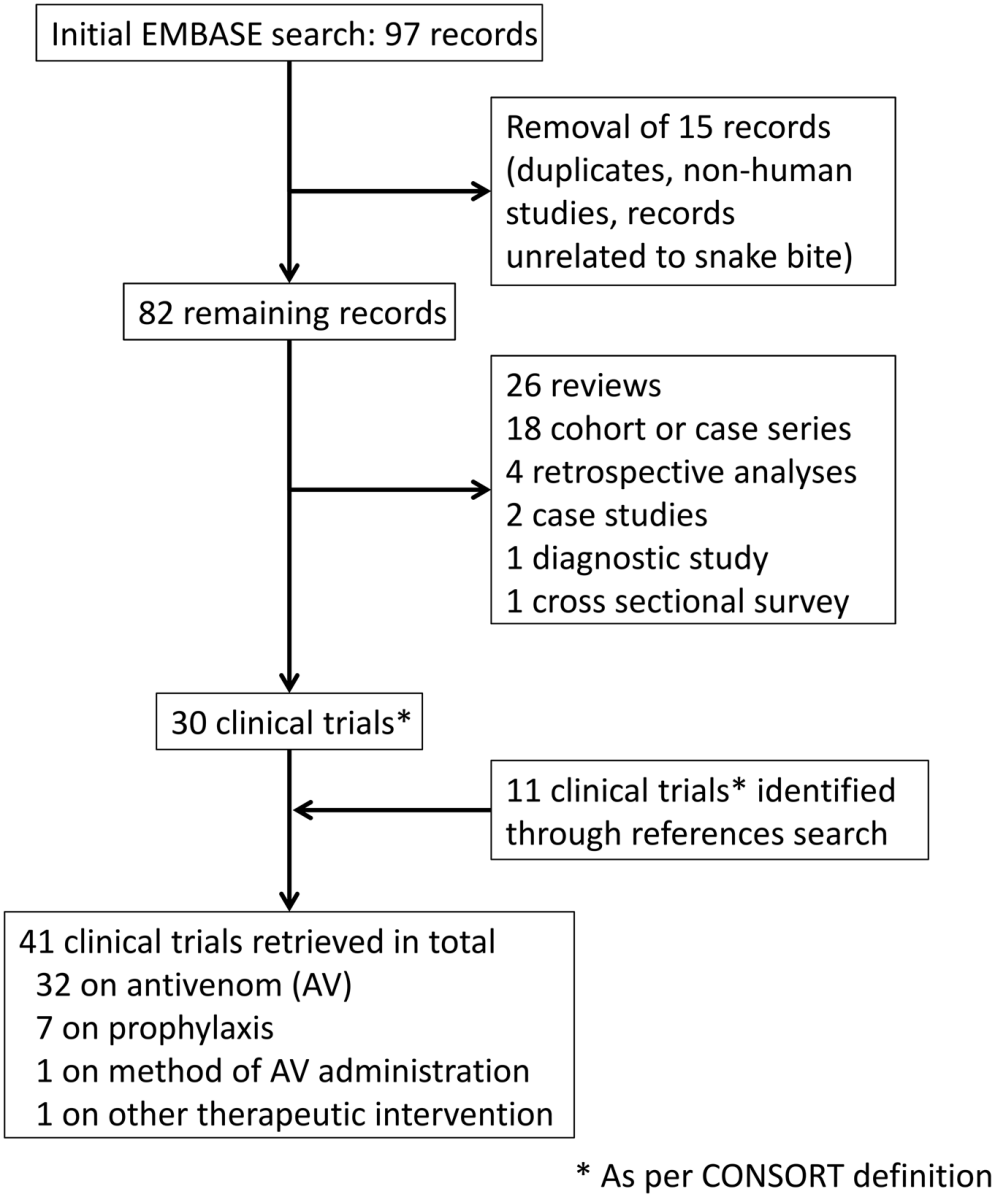
Flow diagram of the selection process used in this study. The search was conducted on 15 September 2014. Merging the search results gave a total of 41 clinical trials investigating the efficacy or safety of snake antivenoms, of which four were active. A total of 36 different antivenoms were investigated (see Table 2). Based on the trial design (Phase I to IV), ten products were considered still “under development,” although development appears to have stalled for most of them. Our search strategy appears robust; a report conducted in 2010 identified a total of 43 randomized controlled trials on snakebite envenoming, 28 of which investigated antivenom properties [11]. We retrieved all except two of these trials [12,51]; the discrepancy could be due to differences in the criteria used to define clinical trials.

The publication database search yielded 97 results (Fig 1). After cleaning, 82 records were retained, of which 30 had a design consistent with clinical trials. The remainder included 26 reviews or commentaries, 18 cohorts or cases series, four retrospective analyses of medical records, two case studies, one diagnostic study, and one cross-sectional survey. A search of references yielded an additional 11 reports of clinical trials. Of the 41 clinical trials thus identified, 32 investigated antivenom as an intervention. The locations of the 32 studies were Latin America (Brazil *n* = 3, Columbia *n* = 5, Ecuador *n* = 1); Asia (India *n* = 4, Thailand *n* = 5, Sri Lanka *n* = 3, Myanmar *n* = 1, Malaysia *n* = 1); Africa (Nigeria *n* = 5), and US (*n* = 4). 27 were sponsored by a public organization (e.g., university or public hospital). Most trials (*n* = 20) were conducted before 2000, the oldest dated from 1960 [10]. A total of 30 antivenoms were investigated; half were investigated in only one trial.

**Table 2:** List of antivenoms investigated in clinical trials published in peer-reviewed journals or on public registries.

Product name	Other name/product specifications	Manufacturer	Development stage^1^	Target region	Publications	Clinical trials registry number
CroFab	Polyvalent ovine antivenom (Fab) against Crotalid	Protherics	Phase III–IV	North America	[22,24,25]	NCT00303303 NCT00636116 NCT00868309 NCT01864200
Anavip	Polyvalent equine antivenom (Fab2) against Crotalinae (pit viper)	Instituto Bioclon S.A.	Terminated after Phase III	North America	[22]	NCT00868309 NCT00636116
Antivypmin	Polyvalent equine antivenom (Fab2) against Crotalinae (pit viper)	Instituto Bioclon S.A.	Phase III	North America	None	NCT00639951
NA	Polyvalent equine antivenom (Fab2) against North American Coral snakes (*Micrurus*)	University of Arizona	Phase III	North America	None	NCT01337245
Tiger snake antivenom	Monovalent equine (Fab) against *Notechis scutatus*	CSL	Phase III–IV	Australia	None	ACTRN12611000588998
Taipan antivenom	Monovalent equine (Fab) against *Oxyuranus scutellatus*	CSL	Phase I–II	Australia	None	ACTRN12612001062819
Antibotropico IVB		Instituto Vital Brazil	Phase II	Latin America	[26]	None
Antibotropico Butantan	Polyvalent equine antivenom against *Bothrops* species	Instituo Butantan	Phase II–III	Latin America	[26–29]	None
Antibotropico FUNED		Fundação Ezequiel Dias	Terminated	Latin America	[26]	None
Antibotropico-laquetico Butantan	Bothrops-Lachesis polyvalent equine antivenom	Instituo Butantan	Phase II	Latin America	[30]	None
Antiofiodico botropico polivalente	Polyvalent equine antivenom (IgG) against *Bothrops asper*, *Bothrops atrox*, and *Bothrops xanthogrammus*	Instituto Nacional de Higiene y Medicina Tropical "Leopoldo Izquieta Pérez"	Phase II–III	Latin America	[28]	None
Monovalent *B*. *atrox* equine antivenom		Instituto Clodomiro Picado	Terminated	Latin America	[31,32]	None
Monovalent *B*. *atrox* equine antivenom		Instituto Nacional de Salud	Terminated	Latin America	[29]	None
*B*. *atrox–Lachesis* antivenom	Polyvalent equine antivenom (IgG) against *B*. *atrox* and *Lachesis muta muta*	Fundação Ezequiel Dias	Terminated	Latin America	[30]	None
Polyvalent Antivenom	Polyvalent equine antivenom (IgG) against *B*. *asper*, *Crotalus durissus*, and *L*. *muta*	Instituto Nacional de Salud	?	Latin America	[28]	None
Polyvalent antivenom ICP	Polyvalent equine antivenom (IgG or Fab2) *against B*. *asper*, *Crotalus simus*, and *Lachesis stenophrys*	Instituto Clodomiro Picado (University of Costa Rica)	Phase II	Latin America	[31–34]	None
EchiTab	Monovalent ovine antivenom (Fab) against *Echis oscellatus*	Therapeutic Antibodies/Micropharm	?	Sub-Saharan Africa	[35]	None
EchiTab Plus	Polyvalent equine antivenom against *Bitis arietans*, *E*. *oscellatus*, and *Naja nigricollis*	Instituto Clodomiro Picado (University of Costa Rica)	Phase I–II	Sub-Saharan Africa	[23,36]	ISRCTN01257358
EchiTab G	Monovalent antivenom (IgG) against *E*. *oscellatus*	Micropharm	Phase I–II	Sub-Saharan Africa	[23,36]	ISRCTN01257358
EgyVac antivenom	Equine polivalent antivenom against *B*. *arietans*, *E*. *oscellatus*, and *N*. *nigricollis*	Vacsera Ltd	Terminated after Phase I	Sub-Saharan Africa	[36]	None
Ipser Africa Antivenom	Polyvalent equine (Fab2) antivenom against *B*. *arietans*, *Bitis gabonica*, *Echis leucogaster*, *N*. *nigricollis*, *Naja haje*, *Naja melanoleuca*, *Dendroaspis viridis*, *Dendroaspis jamesoni*, and *Dendroaspis augisticeps*	Institut Pasteur	?	Sub-Saharan Africa	[35]	None
Monospecific antivenom against *E*. *oscellatus*		Institut Pasteur	?	Sub-Saharan Africa	[37,38]	None
SAIMR Echis antivenom	Monovalent equine antivenom (IgG or Fab2) against *Echis carinatus* / *ocellatus*	South African Vaccines Producer	?	Sub-Saharan Africa	[38]	None
North and West African polyvalent antivenom (*Echis*, *Bitis*, *Naja*)		Behningwerke	?	Sub-Saharan Africa	[37,38]	None
Malayan pit viper antivenom	Monovalent equine antivenom against *Calloselasma rhodostoma*	Queen Saovabha Memorial Institute	Phase I–II	South East Asia	[11,39–41]	None
Malayan pit viper antivenom	Monovalent caprine antivenom against *C*. *rhodostoma*	Twyford Pharmaceutical	Phase I–II	South East Asia	[39–41]	None
Malayan pit viper antivenom	Monovalent equine antivenom against *C*. *rhodostoma*	Thai Government Pharmaceutical Organisation	Phase I–II	South East Asia	[39–41]	None
Monocellate cobra antivenom	Monovalent equine antivenom against aja. *kaouthia*	Queen Saovabha Memorial Institute	?	South East Asia	[42]	None
Green pit viper antivenin (QSMI)	Polyvalent equine antivenom (Fab2) against green pit vipers	Queen Saovabha Memorial Institute	Phase I–II	South East Asia	[41,43]	None
*B*. *multicinctus* and *B*. *candidus* antivenom	Polyvalent equine antivenom (Fab2) against *Bungarus multicinctus* and *Bungarus candidus*	Vietnam Poison Control Center, Hanoi Medical University	Phase I–II	South East Asia	[21]	NCT00811239
Monospecific antivenom against *D*. *russelii*		Myanmar Pharmaceutical Factory	?	South East Asia	[44]	None
ProlongaTab	Monovalent ovine antivenom (Fab) against *Daboia russelii*	Therapeutic Antibodies Inc	Terminated	South Asia	[45,46]	None
SII Polyvalent ASV IP	Polyvalent equine antivenom (Fab2) against *Naja naja*, *E*. *carinatus*, *D*. *russelii* and *Bungarus caeruleus*	India Serum Institute	?	South Asia	[47–49]	None
Snake antivenin IP	Polyvalent equine antivenom (Fab2) against *N*. *naja*, *E*. *carinatus*, *D*. *russelii* and *B*. *caeruleus*	Haffkine Biopharmaceutical Corporation Ltd	Phase II	South Asia	[45,46,50,51]	None
Snake venom anti-serum	Polyvalent equine F(ab)2 against *B*. *caeruleus*, *N*. *naja*, *D*. *russelii* and *E*. *carinatus*	VINS bioproducts	Phase II	South Asia	None	SLCTR/2010/006 NCT01284855
Snake venom antiserum	Polyvalent equine F(ab)2 against *B*. *caeruleus*, *N*. *naja*, *D*. *russelii* and *E*. *carinatus*	Bharat Serum and Vaccines Ltd	Phase II	South Asia	None	SLCTR/2010/006

^1^ Not all publications mentioned the trial phase, and development status was established based on trial design, primary objectives, and number of subjects. This classification, though, bears some limitations, especially with regards to snake antivenoms development, in which Phase I with healthy volunteers are generally not conducted.

## Urgent Need for More Research

Our results highlight the paucity of adequately conducted clinical trials and corroborate previous findings on the scarcity of safe, effective, and quality-assured snake antivenoms [4]. Comparison with dengue fever, which has a similar burden (11.97 Disability-Adjusted Life Years (DALYs) per 100,000 [4.99–20.46] versus venomous animal contacts 39.62 DALYs per 100,000 [22.46–69.74]) [13], is particularly revealing. In 2011, of 79 identified trials on dengue fever, 27 were recruiting patients, with six new products in development [14]. By contrast, the research pipeline for snakebite remains desperately dry, despite numerous calls for action [15–17].

## Antivenoms in Sub-Saharan Africa

To determine how many antivenom products are currently available in sub-Saharan Africa, we searched WHO “Venomous snakes and antivenoms database” and held bilateral discussions with snakebite experts and pharmaceutical companies. We found that 12 antivenom products were commercially available in sub-Saharan countries as of September 2014 (Table 3), only three of which had been tested in at least one clinical trial, and many of which may lack efficacy [18].

**Table 3:** Available snake antivenom products in sub-Saharan Africa, as of September 2014.

Product	Company	Country of production
Antivipmyn-Africa	Instituto Bioclon/Silanes	Mexico
ASNA-C	Bharat Serums and Vaccines	India
ASNA-D	Bharat Serums and Vaccines	India
EchiTabG	MicroPharm	United Kingdom
EchiTabPlus	Instituto Clodomiro Picado	Costa Rica
Fav-Afrique	Sanofi Pasteur	France
Inoserp PanAfrica	Inosan	Spain
SAIMR Boomslang antivenom	South African Vaccine Producers	South Africa
SAIMR Echis antivenom	South African Vaccine Producers	South Africa
SAIMR Polyvalent Snake antivenom	South African Vaccine Producers	South Africa
Snake Venom Antiserum (Pan-African)	VINS Bioproducts	India
Snake venom antiserum Echis ocellatus	VINS Bioproducts	India

### Case study: The MSF experience in Central African Republic

The experience of MSF in CAR suggests that there are indeed significant variations in the efficacy of antivenoms against African snake venoms. MSF has been using Fav-Afrique to manage patients presenting with features of snakebite envenoming in Paoua, CAR, since 2008. In the first half of 2013, Fav-Afrique was temporarily unavailable, and an alternative product was identified, directed against the venoms of 11 species of African snakes, including *E*. *ocellatus*. This antivenom was used for six months, with the same criteria for therapy as for Fav-Afrique. Although a methodologically sound study could not be conducted, a retrospective analysis of MSF medical records showed that the case fatality rate increased from 0.47% (three of 644 treated patients) with Fav-Afrique [9] to 10% (five of 50 treated patients) with the alternative antivenom. While more than 80% of patients were successfully treated with only one dose of Fav-Afrique, more than 60% treated with the alternative antivenom (31 of 50) required more than one dose to control envenoming. Worryingly, the first dose of the alternative antivenom was not able to alleviate spontaneous bleeding at admission in ten of 13 patients, and the administration of additional doses was required. These field data need cautious interpretation. However, they echo findings on the availability of ineffective and potentially harmful antivenoms in sub-Saharan Africa and support the conclusion that post-marketing surveillance is crucial [18]. They also call for a more robust and systematic evaluation of marketed products by regulatory authorities in the affected countries.

## The Way Forward

Sanofi Pasteur urgently needs to disclose its plan to mitigate the negative impact of the decision to stop producing Fav-Afrique. Over the longer term, the multi-component strategy described by the Global Snakebite Initiative must be fully financed [19]; both innovations for better products and interventions and access to quality care need to be enhanced. The vast majority of the trials that we identified were sponsored by public organizations. The snakebite antivenom market so far appears poorly lucrative, unpredictable, and fragmented, hindering investment from pharmaceutical companies [4]. A major donor needs to step in, provide support, and, importantly, encourage existing global health initiatives, such as Drugs for Neglected Diseases initiative (DNDi), the Global Alliance for Vaccine and Immunization (GAVI)-Alliance, or the European and Developing Countries Clinical Trials Partnership (EDCTP), to extend their remits to life-saving treatments for snakebites. Finally, WHO should fully include snakebite envenoming in its list and programme of NTDs, support national regulatory authorities in performing adequate evaluations of existing antivenom products, and establish partnerships for access to existing and future antivenoms. Snakebite envenoming has been a most neglected disease for far too long.
